# Efficient secretory production of recombinant proteins in microalgae using an exogenous signal peptide

**DOI:** 10.3389/fmicb.2025.1603204

**Published:** 2025-06-18

**Authors:** Trang Thi Le, Quynh-Giao Tran, Su-Bin Park, Hyang Ran Yoon, Dong-Yun Choi, Dae-Hyun Cho, Jin-Ho Yun, Hong Il Choi, Hee-Sik Kim, Yong Jae Lee

**Affiliations:** ^1^Cell Factory Research Center, Korea Research Institute of Bioscience and Biotechnology (KRIBB), Daejeon, Republic of Korea; ^2^Department of Environmental Biotechnology, KRIBB School of Biotechnology, University of Science and Technology, Daejeon, Republic of Korea; ^3^Department of Biological Resource Research, Nakdonggang National Institute of Biological Resource, Sangju, Republic of Korea; ^4^Immunotherapy Convergence Research Center, KRIBB, Daejeon, Republic of Korea

**Keywords:** microalgae, synthetic biology, signal peptide, secretory pathway, recombinant protein

## Abstract

Microalgae are promising platforms for recombinant protein production due to their scalability, rapid growth, safety, and sustainability. One strategy to reduce downstream processing costs is to secrete recombinant proteins directly into the culture medium, facilitated by signal peptides (SPs). However, the limited availability of effective SPs has hindered broader applications of this approach in microalgae. In this study, we identified a novel SP from a highly secreted protein of approximately 17 kDa in the culture medium of *Chlorella* sp. HS2. N-terminal sequencing via Edman degradation enabled identification of the corresponding gene, which encodes a hypothetical protein we designated MAPS (Most Abundant Protein in the Secretome). Bioinformatic analyses revealed a functional SP with features consistent with efficient secretory activity. To evaluate its utility, we generated transgenic *Chlamydomonas reinhardtii* strains expressing mCherry fused to this *Chlorella* sp. HS2-derived SP. Compared to two commonly used endogenous SPs from *C. reinhardtii*, the HS2-SP significantly enhanced mCherry secretion, achieving approximately two-fold higher levels in the culture medium. These findings highlight the potential of HS2-SP in improving recombinant protein secretion in *C. reinhardtii*, thereby supporting its application in algal biotechnology and industrial protein production.

## 1 Introduction

Microalgae are emerging as powerful platforms in biotechnology, particularly for the production of food, feed compounds, and high-value products such as polyunsaturated fatty acids and carotenoids (Levasseur et al., 2020). Importantly, microalgae naturally contain high protein levels, ranging from 30 to 70% of dry weight in common species, making them especially attractive for recombinant protein production. For example, *Arthrospira maxima* (60–71%), *Chlorella* sp. (51–58%), *Dunaliella salina* (50–80%), *Chlamydomonas reinhardtii* (48%), *Phaeodactylum tricornutum* (39.6%), and *Nannochloropsis oceanica* (35–44%) have been reported to surpass traditional protein sources such as skim milk (36%), soybean meal (37%), chicken (24%), fish (24%), and peanuts (26%) (Becker, 2007; Tibbetts et al., 2015; Janssen et al., 2022). Algae can produce 2–10 times more biomass per unit of land area than the most productive terrestrial systems (Sayre, 2010). Unlike conventional crops, microalgal cultivation is not limited by growing season or soil fertility, allowing year-round production. Additionally, various cultivation systems, such as open ponds and closed photobioreactors, along with both autotrophic and heterotrophic modes, allow for flexible and scalable production strategies suitable for different environments. For recombinant protein production, genetically engineered microalgae grown in bioreactors offer additional benefits. These include minimizing the risk of pathogen contamination, which is essential for maintaining robust algal growth and ensuring the safe commercialization of therapeutic products, as well as preventing the release of transgenes into the environment. In addition, culture medium recycling further enhances sustainability by reducing water and nutrient loss (Specht and Mayfield, 2014). Several microalgal species, including *C. reinhardtii* and *P. tricornutum*, have already been engineered to produce therapeutic proteins (Mayfield and Franklin, 2005; Rasala et al., 2010; Hempel et al., 2011; Gimpel et al., 2015), vaccines (Sun et al., 2003; He et al., 2007), and valuable industrial enzymes (Georgianna et al., 2013; Lauersen et al., 2013b; Kang et al., 2025). Importantly, the timeframe for algal transformation and scale-up is considerably shorter than that required for higher plants, which may take months to years to produce homozygous seeds (Passricha et al., 2016). Overall, these advantages position microalgae as a promising system for next-generation biomanufacturing.

For recombinant proteins to maintain their function and stability, post-translational modifications (PTMs) are important. Different expression systems offer different capabilities for PTMs, each with its own advantages and challenges. For example, bacterial expression systems often encounter problems with inappropriate PTMs, such as erroneous disulfide bond formation, which can result in non-functional proteins and complicate downstream purification processes (Berkmen, 2012). In contrast, eukaryotic expression systems, such as yeast, fungi, plants, and animals, offer distinct advantages, particularly in their ability to perform glycosylation, an important PTM that affects protein folding, stability, and activity (He et al., 2024). However, these systems also present challenges in recombinant protein production (Beygmoradi et al., 2023). For instance, proteins expressed in yeast can be hyperglycosylated, raising concerns about potential immunogenicity in human and animal applications (Hamilton and Gerngross, 2007). Filamentous fungi produce high concentrations of proteases, which can degrade heterologous protein products (Ward, 2012). Transgenic animals, although considered one of the most suitable systems for recombinant protein production, face inefficiencies due to the time and cost involved in generating and maintaining transgenic animals, as well as ethical concerns associated with animal experimentation (Dyck et al., 2003). Meanwhile, plant expression systems typically yield low amounts of protein, often limited to approximately 10% of total soluble protein (Mardanova et al., 2017). Moreover, there are significant concerns regarding plant-derived proteoglycans, which have been linked to allergic reactions, necessitating the humanization of their natural glycosylation patterns for therapeutic use (Montero-Morales and Steinkellner, 2018). Importantly, most green algae are classified as Generally Regarded as Safe (GRAS), which simplifies the purification and processing of expressed products for many targeted applications (Sproles et al., 2021). Microalgae can efficiently perform PTMs, including glycosylation and disulfide bond formation, and can successfully assemble large, complex, multi-subunit proteins into functional forms (Dehghani et al., 2020).

A major challenge in using microalgae for industrial applications is the high cost of post-harvest processing, which can exceed 50% of the total production cost (Mallick et al., 2016). Hence, secretion of recombinant proteins into the extracellular matrix is emerging as a promising strategy to address this challenge in recombinant protein production (Ramos-Martinez et al., 2017). In eukaryotic systems, protein secretion ensures proper glycosylation, which plays an important role in determining the yield, biological activity, and stability of secreted recombinant proteins (Lingg et al., 2012). Additionally, this process simplifies downstream processing by bypassing the costly and laborious cell lysis procedure (Nikolov and Woodard, 2004). The harvested algal biomass can then be used as a valuable by-product, adding value and extending the productive life of the cells. Despite these advantages, studies on secretion methods in microalgal expression systems are still scarce and need further exploration. For successful secretion of recombinant proteins, the presence of signal peptides (SPs) located at the N-terminus of the protein plays a crucial role (Hegde and Bernstein, 2006). SPs are important in guiding proteins through the entire secretory pathway, involving essential cellular structures such as the endoplasmic reticulum (ER) and Golgi apparatus, ultimately directing them to their final destination, including membranes or extracellular secretion. These short N-terminal sequences, typically 16 to 30 amino acids in length, function in conjunction with recognition motifs to ensure precise protein localization. In *C. reinhardtii*, recombinant protein secretion strategies employ various SPs derived from the host’s secreted protein repertoire or from different species (Table 1). For example, studies have examined several native signal sequences, including those from iron assimilatory protein 1 (FEA1) (Allen et al., 2007), arylsulfatases (ARS1 and ARS2) (Eichler-Stahlberg et al., 2009; Rasala et al., 2012), carbonic anhydrase (CAH1) (Lauersen et al., 2013a), binding protein 1 (BiP1) (Rasala et al., 2014), and metalloprotease gametolysin (Ramos-Martinez et al., 2017). Notably, non-native signal sequences have also been shown to effectively direct recombinant proteins for secretion. In a comprehensive study comparing various secretory SPs for heterologous protein expression in *C. reinhardtii*, the researchers compared SPs from ARS1 and CAH1 proteins with an uncharacterized SP from the ice-binding protein (IBP1), along with six sequences identified through *in silico* methods (Molino et al., 2018). Their findings expand the research portfolio of recombinant protein secretion, not only in this alga but also in microalgae in general. The yield of recombinant protein in *C. reinhardtii* varied widely, from 100 μg L^−1^ to 10 mg L^−1^. In addition, the use of fluorescent proteins and luciferase has proven beneficial in optimizing different aspects of protein secretion, allowing rapid detection and quantification of protein levels. Current research efforts are focused on understanding and improving the recombinant protein secretion capabilities of microalgae to maximize protein yield and efficiency, especially when compared to other established model organisms such as bacteria and yeast.

**Table 1:** Summary of the commonly used signal peptides in *C. reinhardtii*.

Origin	Signal peptide (aa)	Gene identification	Protein size (aa)	Yield	Target protein secretion	References
*Chlamydomonas reinhardti*	MSVGFLVLALGALVVATA	Iron assimilatory protein (*FEA1*)	362	Not mention	FEA1	Allen et al. (2007) In this study
	MHARKMGALAVLAVACLAAVASVAHA	Arylsulfatase 1 (*ARS1*)	654	Not mention	Xylanase, mCherry	Eichler-Stahlberg et al. (2009), Rasala et al. (2012), Molino et al. (2018)
	Not mention	Arylsulfatase 2 (*ARS2*)	507	100 μg L^−1^	Erythropoietin	Eichler-Stahlberg et al. (2009)
	MARTGALLLVALALAGCAQA	Carbonic anhydrase (*CAH1*)	377	84%	Luciferase, mCherry	Lauersen et al. (2013a), Molino et al. (2018)
	MAQWKAAVLLLALACASY	Binding protein 1 (*BiP1*)	656	Not mention	mCherry	Rasala et al. (2014), Molino et al. (2018)
	MPSSSMKLFAALLIACMAQTSMA	Ice-binding protein 1 (*IBP1*)	353	Not mention	mCherry	Raymond et al. (2009), Molino et al. (2018)
	MSLATRRFGAAAALLVAACVLCTAPAWA	Metalloprotease gametolysin	610	1.3 mg L^−1^	Venus	Ramos-Martinez et al. (2017) In this study
	MTLRLAQLALATLGVLLLVLAPMPALS	Possible cell wall protein (*SAD1p*)	4,531	Not mention	mCherry	Molino et al. (2018)
	MRGIIAAYTSATLLALLLVTWLTHSSA	Mitogen-activated protein kinase kinase kinase 7	1,339	Not mention	mCherry	
*Momordica charantia*	MRRAIALGVGLALLGLLLPGSLA	Glycoside-hydrolase-like protein, 1,3-*α*-glucosidase assembly Fact. 4, mitochondrial like	1,641	Not mention	mCherry	
*Volvox carteri*	MARRLLLALALAAVLGLAHA	Prolyl 4-hydroxylas	273	Not mention	mCherry	
*Chlorella* UTEX 395	MAGRITLLLCLCLVAGAAA	Cellulase	8.4 kDa	Not mention	Human granulocyte-colony stimulating factor (hG-CSF)	Shin et al. (2020)
	MKGALLLLLLALAASAAIA	Ras-related RABF1	17.7 kDa			
*Picochlorum renovo*	MKGNLARHSLFALFVAVFLSAAQS	Native protein (008562) pLRD42	Not mention	0.37 mg L^−1^	mCherry	Dahlin and Guarnieri (2021)
*Chlorella* sp. HS2	MRTALLLAVLAACLFAVSA	Unknown protein	139	94%	mCherry	In this study

Previously, we isolated a novel strain, *Chlorella* sp. HS2, from a tidal rock pool near a coastal waterfall on Jeju Island, Republic of Korea. This strain exhibited remarkable tolerance to a wide range of salinities, pH levels, and temperatures, along with impressive biomass productivity under autotrophic, mixotrophic, and heterotrophic conditions (Yun et al., 2019). In this study, we identified a novel SP from this alga and examined its efficacy in facilitating recombinant protein secretion in *C. reinhardtii.* We compared the secretion efficiency of this *Chlorella* sp. HS2-derived SP (hereinafter referred to as HS2-SP) with that of endogenous SPs from the metalloprotease gametolysin and the FEA1 protein, using mCherry fused to each SP. Our findings indicate that HS2-SP has significant potential in enhancing protein secretion in *C. reinhardtii*, which could advance its application in recombinant protein production.

## 2 Materials and methods

### 2.1 Microalgal strains and cultivation conditions

The *Chlorella* sp. HS2 strain was isolated from a tidal rock pool on Jeju Island, Republic of Korea (Yun et al., 2019). The wild-type (WT) strain of *C. reinhardtii* CC-124 [137c] was obtained from the Chlamydomonas Resource Center at the University of Minnesota (USA). All algal cells were cultured in Tris-acetate phosphate (TAP) medium under continuous illumination of 50 μmol photons m^−2^ s^−1^ at 25°C, with shaking at 120 rpm. For each sampling point, cell density was measured by assessing the optical density of 200 μL of cell culture at 680 nm using a Spark^®^ Multimode Microplate Reader (Tecan, Switzerland).

### 2.2 Preparation of secreted proteins from microalgal culture supernatant

To isolate secreted proteins from the microalgal culture supernatant, 10 mL of supernatant was collected from the culture on day 10 by centrifugation at 12,000 rpm for 5 min. The supernatant was then filtered through a hydrophilic PVDF membrane with a pore size of 0.2 μm (Advantec, Japan) to remove residual cells. Subsequently, three volumes of ice-cold acetone (Sigma-Aldrich, USA) were added to the solution, and the mixture was stored overnight at −20°C to allow protein precipitation. The proteins were then collected by centrifugation at 15,000 rpm for 10 min, and the supernatant was carefully discarded. The resulting pellet was air-dried for 30 min and resuspended in 100 μL of 1 × phosphate-buffered saline (PBS) (LPS Solution, Republic of Korea).

To visualize secreted proteins by sodium dodecyl sulfate-polyacrylamide gel electrophoresis (SDS-PAGE), concentrated protein samples were heated in 4 × NuPAGE LDS sample buffer (Thermo Fisher Scientific, United States). Samples were then loaded onto Mini-PROTEAN TGX 4–20% (w/v) precast protein gels (Bio-Rad, United States). Electrophoresis was conducted at 100 V using Tris-glycine-SDS buffer (LPS Solution, Republic of Korea), followed by staining with Coomassie Blue R250 (LPS Solution, Republic of Korea) for protein visualization.

### 2.3 Protein sequencing by Edman degradation

Edman degradation was employed to characterize a distinct protein band with a molecular weight of approximately 17 kDa observed in the culture medium of *Chlorella* sp. HS2. For protein identification, proteins were transferred from the SDS-PAGE gel onto a 0.2 μm Trans-Blot Turbo Mini PVDF membrane (Bio-Rad, United States) using the Trans-Blot Turbo Transfer System (Bio-Rad, United States), following the manufacturer’s instructions. The membrane was subsequently stained with Coomassie Blue staining solution. The targeted 17 kDa band was excised and sent for N-terminal sequencing at eMass Analysis Lab (Republic of Korea).

### 2.4 Signal peptide identification

The sequence and location of the secreted protein were determined by BLAST search against the genome of *Chlorella* sp. HS2^1^ using the 10 amino acid sequence obtained through Edman degradation. Gene prediction was performed using the GENSCAN Web Server^2^ to determine the coding sequence of the identified protein. To amplify the target gene from *Chlorella* sp. HS2 cDNA, two forward primers targeting the start codon and one reverse primer were used: MAPS_F1 (5’-ATGTTGGCGGCAGTTTGCGGA-3′), MAPS_F2 (5’-ATGCGGACTGCACTGCTTTTGG-3′), and MAPS_R (5’-TCATGCGCACTCAACGCGG-3′).

Following the identification of the secreted protein, Promoter-2.0^3^ was used to identify potential promoter regions, and SignalP 6.0^4^ was used to predict the presence of a signal sequence. For protein functional annotation, the following bioinformatic tools were used: NCBI’s Conserved Domain Database^5^, PROSITE^6^, and InterPro^7^.

### 2.5 Construction of *Chlorella* sp. HS2 cDNA library

Total RNA was extracted from cells in the exponential growth phase using the PureLink™ RNA Mini Kit (Invitrogen, United States) and treated with DNase I using the RQ1 RNase-Free DNase Kit (Promega, United States), following the manufacturer’s protocol. The concentration and purity of RNA were evaluated by measuring the absorbance at 260 and 280 nm using a NanoPhotometer P330 (Implen, Germany). First-strand cDNA was synthesized from 500 ng of total RNA in a 20 μL reaction using the GoScript™ Reverse Transcription System (Promega, United States) with the supplied oligo(dT) primers. The resulting cDNA served as a template for amplification of the gene encoding the secreted protein.

### 2.6 Plasmid construction

To generate mCherry expression vectors with different signal sequences, the *Chlamydomonas* codon-optimized *mCherry* gene was amplified from the plasmid pBR9-mCherry-Cr (Rasala et al., 2013). *mCherry* without SP was used as a negative control for the protein secretion assay. Three different SPs were employed in this study: the identified SP from *Chlorella* sp. HS2, and two *Chlamydomonas* endogenous SPs from metalloprotease gametolysin (Ramos-Martinez et al., 2017) and FEA1 (Allen et al., 2007). These SPs were fused between the start codon and the *mCherry* sequence using a set of overlapping primers.

For plasmid construction, *mCherry* fragments, with or without SP, were cloned into the pChlamiRNA3int vector (Chlamydomonas Resource Center, United States) downstream of the PSAD-RBCS2_intron1 regulatory region. This cloning step replaced the truncated miRNA_PRECURSOR_CRE-mir1157 sequence with the target gene sequence, resulting in the expression vectors ∆mCherry, Me-mCherry, Fe-mCherry, and XS-mCherry. PCR-amplified fragments were purified and assembled using Gibson Assembly Master Mix (New England Biolabs, United States), following the manufacturer’s instructions. The final plasmid constructs were validated by Sanger sequencing (Cosmogenetech, Republic of Korea). All primers and vectors used for plasmid construction and sequencing are listed in Table 2.

**Table 2:** List of primers used in this study.

Primer name	Sequence (5′ to 3′)	Usage
∆mCherry_insert_F	GTTTCCATTTGCAGCTCGAGATGGTGTCCAAGGGCGAGGAGGA	Cloning
∆mCherry_insert_R	GTCCAGCTGCTGCCATCTAGATTATTACTTGTACAGCTCGTCCATGC	Cloning
∆mCherry_Backbone_F	GTCCAGCTGCTGCCATCTAGATTATTACTTGTACAGCTCGTCCATGC	Cloning
∆mCherry_Backbone_R	TCCTCCTCGCCCTTGGACACCATCTCGAGCTGCAAATGGAAAC	Cloning
Me-mCherry_insert_F1	GGCACTTCTAGTCGCCGCATGCGTGCTATGCACAGCTCCTGCGTGGGCTGTGTCCAAGGGCGAGGAGGA	Cloning
Me-mCherry_insert_F2	GTTTCCATTTGCAGCTCGAGATGTCGCTGGCGACGCGGCGCTTCGGCGCCGCAGCGGCACTTCTAGTCGCCGCAT	Cloning
Me-mCherry_Backbone_R	AAGCGCCGCGTCGCCAGCGACATCTCGAGCTGCAAATGGAAAC	Cloning
Fe-mCherry_insert_F1	TCTCGCTCCCCCAGCATCGCAATTGTCCTCGCGGCGGTCGCCCTCCTGGGCGTCTGCGCGCTGGCCGTGTCCAAGGGCGAGGA	Cloning
Fe-mCherry_insert_F2	GTTTCCATTTGCAGCTCGAGATGTCTCGCTCCCCCAGCAT	Cloning
Fe-mCherry_Backbone_R	ATGCTGGGGGAGCGAGACATCTCGAGCTGCAAATGGAAAC	Cloning
XS-mCherry_insert_F1	TGCCTGTTCGCGGTTTCCGCGGTGTCCAAGGGCGAGGAGGA	Cloning
XS-mCherry_insert_F2	GTTTCCATTTGCAGCTCGAGATGCGGACTGCACTGCTTTTGGCTGTGCTTGCAGCCTGCCTGTTCGCGGTTTCCGC	Cloning
XS-mCherry_Backbone_R	AAAAGCAGTGCAGTCCGCATCTCGAGCTGCAAATGGAAAC	Cloning
mCherry_F	GTGTCCAAGGGCGAGGAGGA	Genotyping
mCherry_R	CTTGTACAGCTCGTCCATGC	Genotyping
NTerminal_check_F	TAGGTGTTGCGCTCTTGACT	Sequencing
MAPS_F1	ATGTTGGCGGCAGTTTGCGGA	Gene identification
MAPS_F2	ATGCGGACTGCACTGCTTTTGG	Gene identification
MAPS_R	TCATGCGCACTCAACGCGG	Gene identification

### 2.7 Microalgal transformation

Transformation of *C. reinhardtii* was performed by electroporation using MAX Efficiency^®^ Transformation Reagent for Algae (Invitrogen, United States). Briefly, cells were cultured in TAP medium until they reached a density of 1 × 10^6^ cells mL^−1^. The culture was harvested by centrifugation at 2,000 rpm for 5 min and washed twice with transformation reagent. The resulting cell pellet was resuspended in the same reagent to a final concentration of 2 × 10^8^ cells mL^−1^. Subsequently, 2 μg of linearized plasmid of each construct was added to 250 μL of cell suspension, followed by incubation on ice for 5 min. The DNA-cell mixture was then transferred to a pre-chilled 0.4 cm gap electroporation cuvette (Bio-Rad, United States). Electroporation was performed using the Gene Pulser Xcell™ Total System (Bio-Rad, United States) under the following conditions: 500 V, 50 μF capacitance, and 800 *Ω* resistance.

Following electroporation, cells were incubated on a clean bench for 15 min before being transferred to 10 mL of TAP medium supplemented with 40 mM sucrose. Cultures were allowed to recover under dim light (10 μmol photons m^−2^ s^−1^) at 25°C with gentle shaking at 50 rpm for 14–16 h. The cells were then plated on selective TAP agar plates containing 25 μg mL^−1^ paromomycin (Sigma-Aldrich, United States) and incubated for 10 days until colonies formed.

### 2.8 PCR analysis of putative transgenic lines

Single green colonies were transferred to liquid medium containing 25 μg mL^−1^ paromomycin and further analyzed for mCherry expression after several rounds of selection. Integration of the transgene into the algal genome was confirmed by colony PCR. Briefly, 20 μL of cell culture from each transformant was incubated with 30 μL of 0.5 mM Ethylenediaminetetraacetic acid (Sigma-Aldrich, United States) at 98°C for 20 min using a PCR machine (Bio-Rad, United States). For PCR analysis, 2 μL of each DNA sample was used in a 25 μL reaction with KOD FX Neo DNA polymerase (Toyobo, Japan). PCR products were visualized by electrophoresis on 1% agarose gels stained with Midori Green (Lubioscience, Switzerland). The primers used were mCherry_F and mCherry_R, as listed in Table 2.

### 2.9 Protein extraction and immunoblotting analysis of mCherry

mCherry protein expression and secretion in *Chlamydomonas* transformants were assessed from both cells and culture supernatants by immunoblotting to detect mCherry. Ten mL of algal culture of each transformant and WT strain was centrifuged at 12,000 rpm to separate the cells from the supernatant. The supernatant samples were filtered through a 0.2 μm filter (Advantec, Japan) and precipitated by adding three volumes of ice-cold acetone, following the procedure described above. To extract proteins from cell pellets, lysates were prepared using RIPA buffer (Thermo Fisher Scientific, United States) supplemented with 10 μL mL^−1^ of protease inhibitor cocktail (Sigma-Aldrich, United States). Protein concentration was analyzed using the Pierce™ BCA Protein Assay Kit (Thermo Fisher Scientific, United States).

Protein separation was performed by loading 30 μg of total protein per sample onto 15% SDS-PAGE gels, running at 100 V. The proteins were then transferred to a PVDF membrane (Bio-Rad, United States) at 300 mA for 1 h. For Western blot analysis, anti-mCherry (1:2000; Abcam, United Kingdom) and anti-α-tubulin (1:5000; Abcam, United Kingdom) antibodies were used as primary antibodies to detect mCherry and α-tubulin, respectively. A mouse secondary antibody (1:10,000; Abcam, United Kingdom) was used for both targets. All antibodies were diluted in Tris-buffered saline containing 0.1% Tween 20 (LPS Solution, Republic of Korea) and 5% (w/v) skimmed milk (Becton Dickinson, United States). Signals were visualized using Amersham ECL Western Blotting Detection Reagent (Cytiva, United States), and images were captured using the Bio-Rad ChemiDoc™ MP system (Bio-Rad, United States).

### 2.10 Fluorescence microplate reader analysis

To analyze mCherry fluorescence, mCherry-expressing transformants from each construct were cultured in 20 mL of TAP medium. Cultures were maintained at 25°C under continuous illumination of 50 μmol photons m^−2^ s^−1^. Cells were sampled and analyzed for mCherry fluorescence using a Spark^®^ Multimode Microplate Reader (Tecan, Switzerland), with an excitation wavelength of 572 nm (with a bandwidth of 9 nm) and an emission wavelength of 610 nm (with a bandwidth of 20 nm) and a gain of 170. For whole culture fluorescence, 200 μL of the cell and supernatant mixture was added to a 96-well black plate with a clear bottom (Corning, United States). To analyze the supernatant, cultures were centrifuged at 12,000 rpm for 2 min, and 200 μL of the supernatant was transferred to the same 96-well plate. The cell pellets were washed three times, resuspended in 200 μL of 1 × PBS buffer, and placed in the same plate for fluorescence analysis.

To quantify secretion efficiency, fluorescence values from the whole culture, supernatant, and cell pellet were first normalized to the optical density at 680 nm (OD_680_). Background fluorescence was corrected by subtracting the normalized fluorescence values obtained from the WT sample. Secretion efficiency was calculated as the ratio of the background-corrected fluorescence from the supernatant to the background-corrected fluorescence from the whole culture, expressed as a percentage, using the following formula:


$$SecretionEfficiency\;\left(\%\right)=\frac{\left(\begin{array}{l} Supernatant-\\ WT\;Supernatant \end{array}\right)}{\left(\begin{array}{l} Wholeculture-\\ WT\;Wholeculture \end{array}\right)}\times 100$$

Where *Supernatant* refers to the fluorescence intensity of the supernatant normalized to the OD_680_ of each transformant; *Whole culture* refers to the fluorescence intensity of the whole culture normalized to the OD_680_ of each transformant; *WT Supernatant* and *WT Whole culture* refer to the fluorescence intensity of the supernatant and whole culture from the WT sample, respectively, both normalized to OD_680_.

### 2.11 Confocal microscopic analysis

Confocal fluorescence imaging of cells from each transformant was conducted using a Zeiss LSM510 meta-laser scanning confocal microscope (Carl Zeiss, Germany), equipped with a 60 × objective and a Nikon camera. The excitation and emission spectra were set at 587/610 nm for mCherry and 655/667 nm for chlorophyll *a*. Image processing was performed using Zeiss LSM510 software (Carl Zeiss, Germany).

### 2.12 Statistical analysis

Data visualization and statistical processing were performed using Excel (Microsoft, United States) and Prism 10 (GraphPad, United States) software. Each experiment included at least three biological replicates. Data are presented as means ± standard deviation (SD), with error bars representing the variability between replicates in each figure. Data statistical significance was analyzed using Student’s *t*-test.

## 3 Results

### 3.1 Identification of a highly abundant secreted protein in the culture medium of *Chlorella* sp. HS2

In previous studies, we isolated a novel *Chlorella* sp. HS2 strain with significant potential for industrial applications (Yun et al., 2019; Lee et al., 2020; Yun et al., 2021; Nayak et al., 2022; Kim et al., 2023; Yun et al., 2024a; Yun et al., 2024b). Interestingly, while working with this non-model alga, we observed a significant concentration of secreted proteins in the culture medium, reaching 4 mg L^−1^ by day 10 of cultivation (Supplementary Table S1). It is worth noting that this algal strain exhibited remarkable resilience, surviving a wide range of conditions, including salinity (0–5% w/v NaCl), pH (3.0–10.5), and temperature (14–46°C), while also achieving high biomass accumulation (Yun et al., 2019). Therefore, rather than attributing the detected proteins to passive release via cell lysis, we hypothesized that *Chlorella* sp. HS2 actively secretes certain proteins into the surrounding environment.

**Figure 1:**
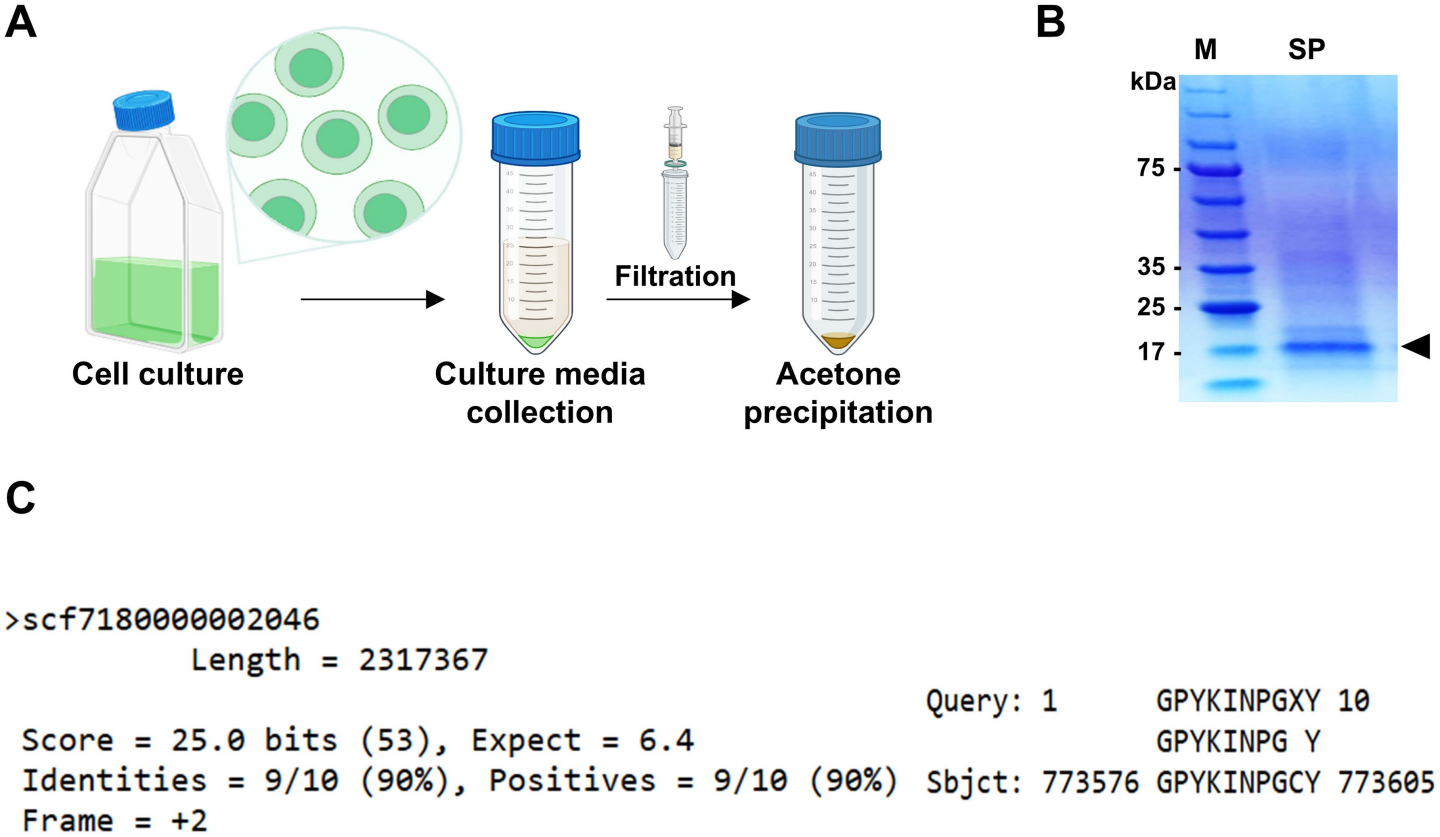
Identification of the most abundant protein in the secretome (MAPS) from *Chlorella* sp. HS2 culture medium. **(A)** Diagram illustrating the sample preparation process for protein analysis. **(B)** Secreted proteins from 10 mL of culture medium were precipitated with acetone and resuspended in 100 μL of 1 × PBS buffer. Samples were separated by SDS-PAGE and visualized by staining with colloidal Coomassie Blue. M, protein marker. SP, secreted proteins. The black triangle indicates the major secreted protein band. **(C)** Alignment of the 10 amino acids obtained by Edman degradation with the *Chlorella* sp. HS2 genomic sequence. Hydroxyproline (X) at cycle 9 was determined to be cysteine (C) based on genomic data.

To investigate this unique protein secretion ability, supernatants from 10-day cultures of *Chlorella* sp. HS2 were filtered and concentrated prior to analysis by SDS-PAGE (Figure 1A). A dominant protein band was observed at approximately 17 kDa, significantly more intense than any other proteins present in the culture medium (Figure 1B). The high abundance of this protein band in *Chlorella* sp. HS2 strongly suggests an active protein secretion mechanism, likely facilitated by a distinct signal sequence. This protein band was subsequently excised for further analysis.

To identify the protein and investigate its functional role, N-terminal sequencing was performed using Edman degradation, a standard method for protein sequence analysis (Miyashita et al., 2001). While SDS-PAGE alone cannot entirely exclude the possibility of co-migrating proteins with similar molecular weights, the well-defined, intense nature of the band, coupled with consistent sequencing results, strongly supports the identification of a single, predominant protein. High-performance liquid chromatography (HPLC) analysis of the Edman degradation cycles yielded a series of phenylthiohydantoin (PTH) amino acids derivatives. The results showed that the N-terminal sequence consisted of 10 amino acids: glycine, proline, tyrosine, lysine, isoleucine, asparagine, proline, glycine, X (tentatively identified as hydroxyproline), and tyrosine (Supplementary Figure S1). The ninth residue was designated as X due to a discrepancy in the relative retention time of its PTH derivative compared to that of the reference material, although hydroxyproline was identified as the most likely corresponding amino acid. The clarity and consistency of the sequencing data, with no evidence of mixed or overlapping signals, support the conclusion that the 17 kDa band corresponds to a single, highly secreted protein.

To gain further insight into the putative secreted protein, the obtained N-terminal sequence (GPYKINPGXY) was subjected to a tBLASTn search against the *Chlorella* sp. HS2 genome. This search resulted in a single high-confidence match at position 773,576 bp in the chromosome region *scf7180000002046*, indicating a high degree of specificity (Figure 1C). Notably, the ninth amino acid, originally designated hydroxyproline, was identified as cysteine based on the genomic sequence. The unique identity of this protein suggests that it may play a specific functional role in *Chlorella* sp. HS2 and is likely associated with a strong SP. Given its remarkable abundance in the *Chlorella* sp. HS2 secretome, we propose naming this protein the Most Abundant Protein in the Secretome (MAPS).

### 3.2 Characterization of the putative signal peptide of MAPS protein

**Figure 2:**
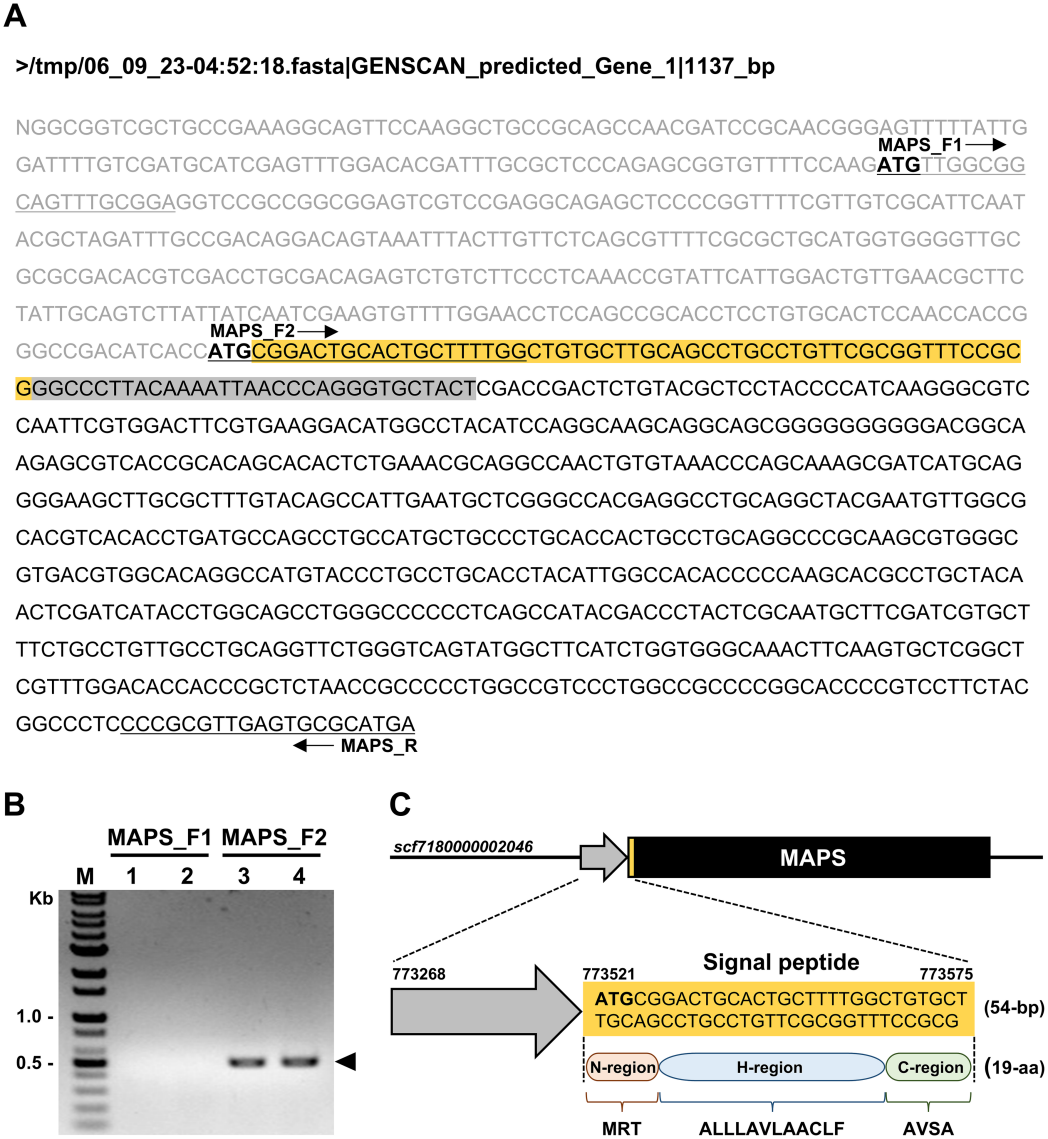
Prediction and validation of the gene encoding the MAPS protein in *Chlorella* sp. HS2. **(A)** Predicted gene sequence generated by the GENSCAN Web Server. Start codons (ATG) are shown in bold. The orange box indicates the predicted signal peptide region, and the gray box represents the coding region corresponding to the N-terminal amino acid sequence identified by Edman degradation. Black arrows indicate the positions of the primers MAPS_F1, MAPS_F2, and MAPS_R. Primer sequences are underlined within the DNA sequence. **(B)** PCR amplification using *Chlorella* sp. HS2 cDNA as the template. Reactions were performed using two forward primers (MAPS_F1 and MAPS_F2) and a reverse primer (MAPS_R) at two annealing temperatures. Lanes: (1) MAPS_F1 and MAPS_R at 60°C; (2) MAPS_F1 and MAPS_R at 65°C; (3) MAPS_F2 and MAPS_R at 60°C; (4) MAPS_F2 and MAPS_R at 65°C. M, DNA marker. The arrow indicates the PCR amplicon corresponding to the MAPS gene. **(C)** Schematic representation of the predicted MAPS gene within the chromosomal region *scf7180000002046*. MAPS refers to the Most Abundant Protein in the Secretome. The black box represents the full-length gene, including exons and introns. The predicted signal peptide is shown in both the nucleotide and translated amino acid sequences.

To identify the DNA sequence encoding the MAPS protein, we analyzed a genomic region spanning 500 nucleotides upstream (toward the 5′ end) and 4,000 nucleotides downstream (toward the 3′ end) of the GPYKINPGCY sequence within the chromosomal region *scf7180000002046*. Since this region was unannotated in the genome browser, we used the GENSCAN program to predict potential genes. Among the two predicted sequences, only one, designated GENSCAN_predicted_Gene_1|1137_bp, contained the 10 amino acids identified through Edman degradation sequencing (Figure 2A). To validate this predicted gene, PCR amplification was performed using cDNA as the template and two forward primers (MAPS_F1 and MAPS_F2), each targeting a different upstream start codon (ATG) (Figure 2A). Notably, only the MAPS_F2 primer, which targets the second start codon, yielded a single, distinct band of 417 bp, confirming the expression of the predicted gene (Figure 2B). The amplified DNA fragment was subsequently purified and subjected to Sanger sequencing to determine its nucleotide sequence.

Functional annotation of the resulting sequence using NCBI’s Conserved Domain Database, PROSITE, and InterPro revealed no conserved domains or known functional motifs. A BLAST search showed that the MAPS protein shares sequence similarity only with hypothetical proteins from other green algal species, including *Micractinium tetrahymenae* and *Micractinium* sp. CCAP 211/92, both closely related to *Chlorella* sp. within the Chlorellaceae family. These homologs also lack functional annotation. Based on these results, the MAPS protein is currently classified as hypothetical, and its specific biological function remains unknown.

Further analysis using SignalP 6.0 revealed the presence of a signal sequence at the N-terminus of the MAPS protein, with a high confidence score of 0.999806. A predicted cleavage site was identified between alanine (position 19) and glycine (position 20) (Supplementary Figure S2). The identified HS2-SP consists of 19 amino acid residues (MRTALLLAVLAACLFAVSA) and exhibits typical SP features, including three distinct regions: a positively charged N-region, a central hydrophobic H-region, and a C-region containing a cleavage site for signal peptidase (Figure 2C). The N-region, represented by the initial MRT residues, contains a positively charged arginine (R) that facilitates interaction with the negatively charged ER membrane, ensuring accurate targeting. The H-region (ALLLAVLA), which is rich in hydrophobic residues, especially leucine (L) and alanine (A), plays an important role in membrane integration. Finally, the C-region, defined by the terminal AVSA residues, contains a predicted cleavage site that enables signal peptidase to process the HS2-SP, facilitating the release of the mature protein into the ER lumen or extracellular space (Guo et al., 2018). This well-structured sequence confirms its function as an efficient SP in directing the MAPS protein to the secretory pathway. These findings support our initial hypothesis, confirm the secretory nature of the MAPS protein, and highlight its potential biological significance.

### 3.3 Generation of mCherry-secreting *C. reinhardtii* strains

**Figure 3:**
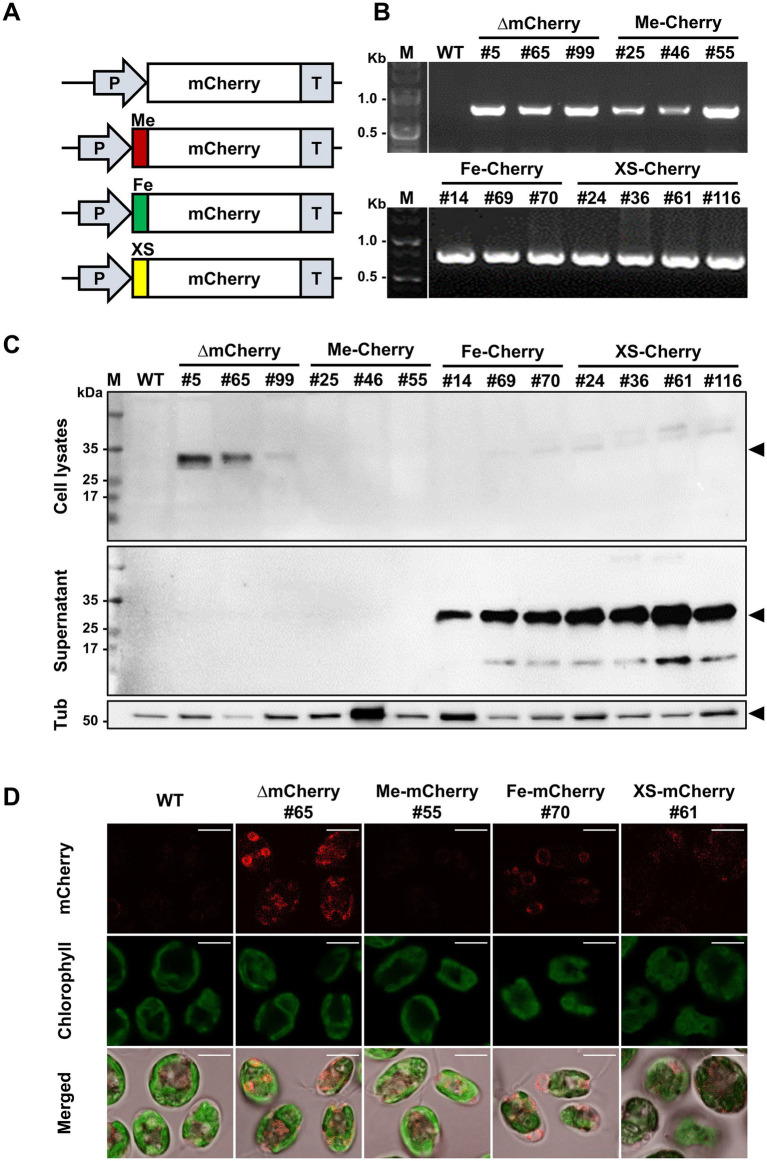
Generation of *Chlamydomonas* transgenic strains for mCherry secretion. **(A)** Schematic diagram of the mCherry expression vector: without a signal peptide (∆mCherry), with signal peptides from the endogenous metalloprotease gametolysin (Me-mCherry) or iron assimilatory protein 1 (Fe-mCherry), and with the putative signal peptide from the *Chlorella* sp. HS2 MAPS protein (XS-mCherry). **(B)** Genotyping of representative transgenic strains for each construct by PCR to confirm *mCherry* gene insertion. M, DNA marker. **(C)** Western blot analysis of cell lysates (harvested cells) and supernatants (culture media) to confirm mCherry expression and secretion. An anti-mCherry antibody was used to detect mCherry (~28 kDa). An anti-tubulin antibody was used to detect tubulin (Tub), a housekeeping protein (~50 kDa). M, protein marker. **(D)** Confocal microscopic images of wild-type (WT), ∆mCherry #65, Me-mCherry #55, Fe-mCherry #70, and XS-mCherry #61 strains showing mCherry expression and localization in *C. reinhardtii*. Scale bar, 5 μm.

To investigate the ability of HS2-SP to direct the secretion of recombinant proteins, the identified HS2-SP was fused to the N-terminus of the mCherry protein, resulting in a vector construct designated XS-mCherry. This plasmid vector contained two expression cassettes: one encoding the mCherry gene and the other carrying an antibiotic resistance marker (paromomycin). As a control, a vector construct expressing mCherry without SP (∆mCherry) was also generated. Additionally, two plasmid vectors expressing mCherry with endogenous SPs from *C. reinhardtii*, specifically the gametolysin SP (Me-mCherry) and the FEA1 SP (Fe-mCherry) (Allen et al., 2007), were constructed for comparison purposes (Figure 3A). The plasmids were subsequently introduced into *C. reinhardtii*, and successful integration was confirmed by PCR using genomic DNA extracted from the transformed cell lines. Following transformation, three independent transgenic lines were selected from each of the ∆mCherry (lines #5, #65, and #99), Me-mCherry (lines #25, #46, and #55), Fe-mCherry (lines #14, #69, and #70), and four lines from the XS-mCherry construct (lines #24, #36, #61, and #116) were selected. These representative lines demonstrated genetic and phenotypic stability over time, as confirmed by colony PCR (Figure 3B), and were maintained on selective plates containing paromomycin prior to further analysis.

To determine the efficiency of mCherry secretion into the culture medium, transgenic lines were cultured in TAP medium, and Western blot analysis was performed on both cell pellets and supernatants. The WT *C. reinhardtii* strain CC-124 served as a negative control for mCherry expression (Figure 3C). In ∆mCherry strains, the protein was detected exclusively in cell lysates, consistent with its expected molecular weight of 28 kDa. In contrast, analyses of the Fe-mCherry and XS-mCherry lines revealed only trace amounts of mCherry in cell lysates, while a significant proportion was present in the culture media, indicating successful secretion. Notably, the accumulation patterns of mCherry in these lines showed a slightly higher molecular weight, with an additional band observed in both the cell pellets and culture media (Figure 3C). This shift in molecular weight may indicate incomplete cleavage of the SP or potential PTMs, such as glycosylation or phosphorylation (Ramos-Martinez et al., 2017). Among the three SP constructs tested, the Me-mCherry lines showed no detectable protein expression, suggesting possible challenges during protein expression. This highlights the complex interplay of factors influencing recombinant protein expression and secretion in *C. reinhardtii*.

In addition to molecular expression analysis, determining the subcellular localization of a recombinant protein is crucial for assessing its secretion efficiency. Confocal laser scanning microscopy was used to visualize the localization patterns of mCherry in representative transgenic lines (∆mCherry #65, Me-mCherry #55, Fe-mCherry #70, and XS-mCherry #61) (Figure 3D). Among these lines, ∆mCherry #65 exhibited strong cytoplasmic fluorescence, indicating active intracellular mCherry expression. In contrast, both Fe-mCherry #70 and XS-mCherry #61 showed weak and punctate cytoplasmic fluorescence, consistent with Western blot results and suggesting that only a small amount of mCherry was retained inside the cells (Figure 3D). Notably, no fluorescence signal was detected in the Me-mCherry #55 line. Taken together, these results demonstrate that the putative SP from the *Chlorella* sp. HS2 MAPS protein functions comparably to the endogenous FEA1 SP in mediating the secretion of recombinant mCherry protein into the culture medium in *C. reinhardtii*.

### 3.4 Efficiency of mCherry protein secretion in transgenic strains

**Figure 4:**
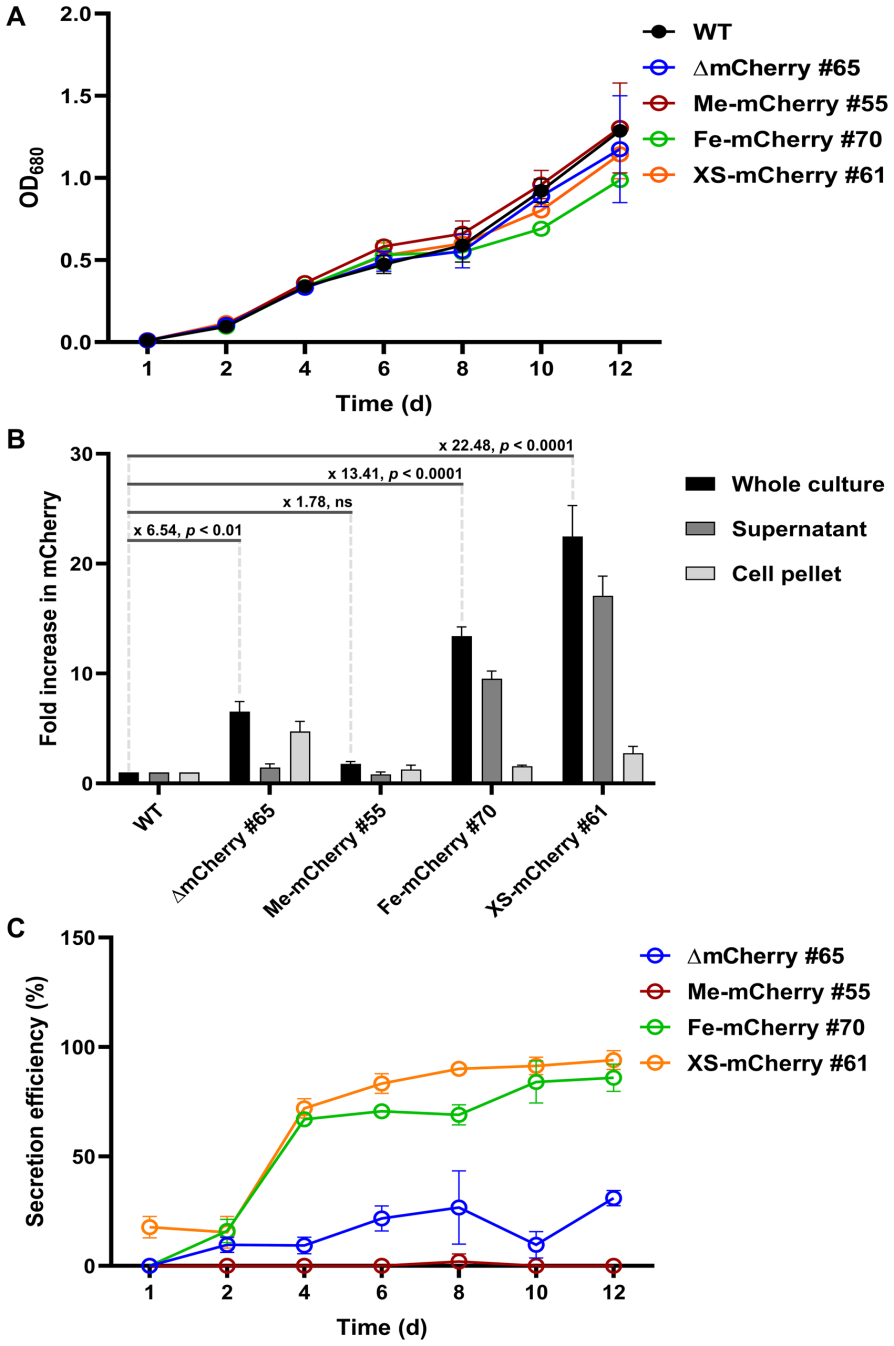
Secretion efficiency of mCherry protein in transgenic lines. **(A)** Growth curves of transgenic lines and the parental wild-type (WT) strain under continuous illumination of 50 μmol photons m^−2^ s^−1^ at 25°C. **(B)** Fold increase in mCherry fluorescence in day-10 cultures. Fluorescence was measured from whole cultures, supernatants (culture media), and cell pellets. Fold changes are showed with statistical significance, as determined by Student’s *t*-test. **(C)** Secretion efficiency was calculated as the percentage of fluorescence in the supernatant relative to the whole culture, with background fluorescence from the WT subtracted. mCherry fluorescence values were normalized to OD_680_. Values represent the average of *n* = 3 biological replicates from a single clonal isolate. Error bars represent standard deviation.

Western blot results indicated that the mCherry protein was successfully secreted into the culture medium by HS2-SP. To quantify both intracellular and extracellular levels of mCherry, a fluorescence plate reader assay was performed, with the WT strain serving as a negative control to establish the baseline level of autofluorescence. No significant difference in growth rate was observed between WT and transgenic lines, indicating that the genetic modifications did not impair cell proliferation (Figure 4A). Fluorescence measurements of the whole culture on day 10 of cultivation revealed significant differences in mCherry levels among the transgenic lines. Specifically, ∆mCherry #65, Me-mCherry #55, Fe-mCherry #70, and XS-mCherry #61 showed 6.54-, 1.78-, 13.41-, and 22.48-fold increases, respectively, compared to WT. The supernatants of both ∆mCherry #65 and Me-mCherry #55 exhibited fluorescence levels comparable to WT (1.0 versus 0.8), consistent with the Western blot results showing no detectable protein bands in their culture supernatants. In contrast, the supernatant of Fe-mCherry #70 showed a 9.5-fold increase in mCherry fluorescence compared to WT, while XS-mCherry #61 exhibited the highest level, with a 17.08-fold increase compared to WT and a 1.8-fold increase compared to Fe-mCherry #70, highlighting its superior secretion efficiency. These findings are further supported by Western blot analysis, where protein bands from XS-mCherry lines were significantly stronger than those of the other constructs (Figure 3C).

Analysis of cell pellets showed that only ∆mCherry #65 exhibited a significant fluorescence increase (4.7-fold over WT), indicating intracellular accumulation likely due to the absence of a SP required for secretion (Figure 4B). To evaluate protein secretion efficiency, background fluorescence from WT samples was subtracted from those expressing mCherry. Secretion efficiency was then calculated as the percentage of supernatant fluorescence relative to that of the whole culture. As expected, ∆mCherry #65 line showed no detectable secretion due to the lack of a functional SP. In contrast, the secretion efficiency of Fe-mCherry #70 and XS-mCherry #61 increased during cultivation, with a sharp rise from day 2 to day 4. Until day 12, secretion efficiency rose from approximately 67 to 94% for both strains (Figure 4C). Overall, these results demonstrate that both the endogenous SP from the FEA1 protein and the HS2-SP confer high secretory capability, with HS2-SP showing slightly higher efficiency during the active growth phase.

## 4 Discussion

Green microalgae are increasingly recognized as valuable platforms for recombinant protein production across a variety of commercial applications, particularly the expression of complex proteins requiring PTMs such as disulfide bond formation (Gong et al., 2011). This potential is exemplified by the development of algae-based vaccines, which have demonstrated efficacy against several infectious diseases, including classical swine fever, foot and mouth disease, hepatitis B, and white spot syndrome virus (Rasala and Mayfield, 2015). In addition, the ability of microalgae to secrete recombinant proteins presents a notable advantage by simplifying downstream processing and enhancing product recovery, thereby contributing to more cost-effective biomanufacturing processes (Ramos-Martinez et al., 2017). Among green microalgae, *C. reinhardtii* has been the most extensively studied model due to its well-established genetic tools and demonstrated ability to express and secrete recombinant proteins into the extracellular environment (Allen et al., 2007; Ramos-Martinez et al., 2017; Molino et al., 2018). In this context, SPs play a crucial role in directing intracellular trafficking, determining protein localization, and facilitating secretion. By targeting proteins to the ER and secretory pathway, SPs influence not only secretion efficiency but also protein folding and yield, critical parameters for biotechnological applications. Several endogenous and heterologous SPs have been successfully applied in *C. reinhardtii* to enhance secretion efficiency (Table 1). While *C. reinhardtii* remains the primary model, recent comparative analyses involving its close relative *C. incerta* have further underscored the importance of optimizing SPs in microalgal systems (Kang et al., 2025).

Building on this, our study identified a unique secreted protein of approximately 17 kDa, designated MAPS, in the secretome of *Chlorella* sp. HS2. The high band intensity observed for this protein suggests a highly efficient secretion mechanism, likely driven by a potent SP. The SP derived from the MAPS protein exhibited superior secretion efficiency when fused to the mCherry reporter protein, outperforming endogenous SPs in *C. reinhardtii*, including that of FEA1. Conversely, although the gametolysin SP has previously been shown to support the secretion of Venus protein at yields up to 1.3 mg L^−1^, our Me-mCherry lines incorporating this SP failed to produce detectable mCherry both intracellularly and extracellularly. This discrepancy is likely due to transgene silencing, a well-documented challenge in nuclear transgene expression in *C. reinhardtii* (Cerutti et al., 1997).

Earlier studies utilizing *C. reinhardtii* CC-1883 and the transgene-optimized UVM4 strain reported notable improvements in recombinant protein production when using optimized SPs. For instance, the native SP from *C. reinhardtii* CAH1 increased yields by up to 84% compared to the *Gaussia* luciferase SP (Lauersen et al., 2013a; Molino et al., 2018). In comparison, HS2-SP achieved secretion efficiencies of up to 94%, indicating superior performance over previously reported SPs. These results strongly support the potential of HS2-SP to enhance recombinant protein secretion in microalgal expression systems. The effectiveness of cross-species SPs depends on their compatibility with the host’s secretory pathway and protein processing mechanisms (Allet et al., 1997). Nevertheless, numerous studies have demonstrated the successful application of cross-species SPs in diverse eukaryotic systems, including microalgae. For example, SPs derived from glycoside hydrolase-like proteins from *Momordica charantia* and prolyl 4-hydroxylase from *Volvox carteri* have been shown to enhance secretion in *C. reinhardtii* (Molino et al., 2018). Collectively, these findings highlight the value of exogenous SPs, such as HS2-SP, in optimizing recombinant protein production in microalgae. The superior performance of HS2-SP compared to endogenous SPs may be attributed to several factors. Sequence analysis revealed that HS2-SP comprises a clearly defined N-region, H-region, and C-region, key elements for efficient targeting to the secretory pathway. The pronounced hydrophobicity of the H-region likely facilitates effective membrane integration, while the well-defined C-region promotes precise cleavage by signal peptidase. Fluorescence microscopy and Western blot analyses confirmed effective secretion of XS-mCherry, with minimal intracellular accumulation, indicating that HS2-SP effectively directed the recombinant protein for secretion. Additionally, the higher apparent molecular weight of secreted mCherry suggests the possibility of PTMs, which may also contribute to the observed increased secretion efficiency. Overall, our data suggest that HS2-SP not only enhances secretion but also mitigates common limitations associated with endogenous SPs, such as intracellular retention.

Optimizing both endogenous and exogenous SPs offers a promising strategy to improve the yield and scalability of recombinant protein production in microalgae. Despite the promising outcomes observed in this study, several limitations remain. Although HS2-SP exhibited high secretion efficiency in *C. reinhardtii*, its performance in other microalgal species has yet to be evaluated. Future research should assess the cross-species utility of HS2-SP to determine whether its efficacy is host-specific. Additionally, optimization of culture conditions, including pH, temperature, and nutrient levels (e.g., NH_4_Cl concentration), could further enhance SP performance (Pessoa et al., 2025). Studying the transcriptional regulation of MAPS gene may provide insights into its functional roles and elucidate the interrelationship between transcription, translation, and protein secretion. Moreover, modifying secretion constructs with enhancers or fusion tags may further improve yields (Ramos-Martinez et al., 2017). A deeper understanding of the molecular interactions between HS2-SP and the *C. reinhardtii* secretory machinery could reveal new mechanistic insights. Lastly, expanding the search for potential candidate SPs from *Chlorella* sp. HS2 or related species could uncover additional high-efficiency SPs for applications in microalgal biotechnology.

## 5 Conclusion

In this study, we identified a novel secreted protein, designated MAPS, from *Chlorella* sp. HS2 and characterized its associated SP, demonstrating its potential in enhancing recombinant protein secretion in *C. reinhardtii*. Secretome analysis revealed a prominent ~17 kDa protein band in the *Chlorella* sp. HS2 culture medium, indicating the presence of an efficient native secretion mechanism. Sequence identification and bioinformatic analyses confirmed that the HS2-SP associated with MAPS protein possesses key features essential for targeting proteins to the secretory pathway. Fusion of the HS2-SP to the mCherry reporter protein and its expression in transgenic *C. reinhardtii* showed significant improvement in secretion efficiency compared to endogenous SPs derived from gametolysin and FEA1. Notably, transgenic lines expressing HS2-SP achieved the highest levels of extracellular mCherry accumulation, with up to a 1.8-fold increase relative to the best-performing endogenous SP. These findings position the HS2-SP as a promising tool for enhancing protein secretion in algal expression systems, with potential applications in large-scale manufacturing. Further research should explore the cross-species applicability of HS2-SP in other microalgal hosts and identify additional high-performance SPs from this industrial microalga.

## Data Availability

All data generated or analyzed during this study are included in this published article. The gene encoding the MAPS protein has been deposited in the NCBI database under accession number XRO64249.1. The genome of Chlorella sp. HS2 used in this study is available at: http://web.seeders.co.kr/hs2/index.php/chl/blast. Additional data supporting the findings of this study are available from the corresponding authors upon reasonable request.

## Footnotes

^1^http://web.seeders.co.kr/hs2/index.php/chl/blast

^2^http://hollywood.mit.edu/GENSCAN.html

^3^https://services.healthtech.dtu.dk/services/Promoter-2.0/

^4^https://services.healthtech.dtu.dk/services/SignalP-6.0

^5^https://www.ncbi.nlm.nih.gov/Structure/cdd/wrpsb.cgi

^6^https://prosite.expasy.org

^7^https://www.ebi.ac.uk/interpro
