# Supplementary material for: Efficient secretory production of recombinant proteins in microalgae using an exogenous signal peptide
Source: Front Microbiol. 2025 Jun 18;16:1603204. doi: 10.3389/fmicb.2025.1603204 (PMC12213679; doi:10.3389/fmicb.2025.1603204)
Supplement: Supplementary file 1 [file Data_Sheet_1.docx]

Supplementary Material

Efficient secretory production of recombinant proteins in microalgae using an exogenous signal peptide

Trang Thi Le^1,2†^, Quynh-Giao Tran^1†^, Su-Bin Park^4^, Hyang Ran Yoon^3^, Dong-Yun Choi^1^, Dae-Hyun Cho^1^, Jin-Ho Yun^1,2^, Hong Il Choi^1,2^, Hee-Sik Kim^1,2*^, Yong Jae Lee^1,2*^

^1^Cell Factory Research Center, Korea Research Institute of Bioscience and Biotechnology (KRIBB), Daejeon 34141, Republic of Korea

^2^Department of Environmental Biotechnology, KRIBB School of Biotechnology, University of Science and Technology, Daejeon 34113, Republic of Korea

^3^Immunotherapy Convergence Research Center, KRIBB, Daejeon 34141, Republic of Korea

^4^Department of Biological Resource Research, Nakdonggang National Institute of Biological Resource, Sangju 37242, Republic of Korea

***Correspondence:**Yong Jae Lee
[leeyj@kribb.re.kr](mailto:leeyj@kribb.re.kr)

Hee-Sik Kim
[hkim@kribb.re.kr](mailto:hkim@kribb.re.kr)

^†^These authors contributed equally to this work and share first authorship


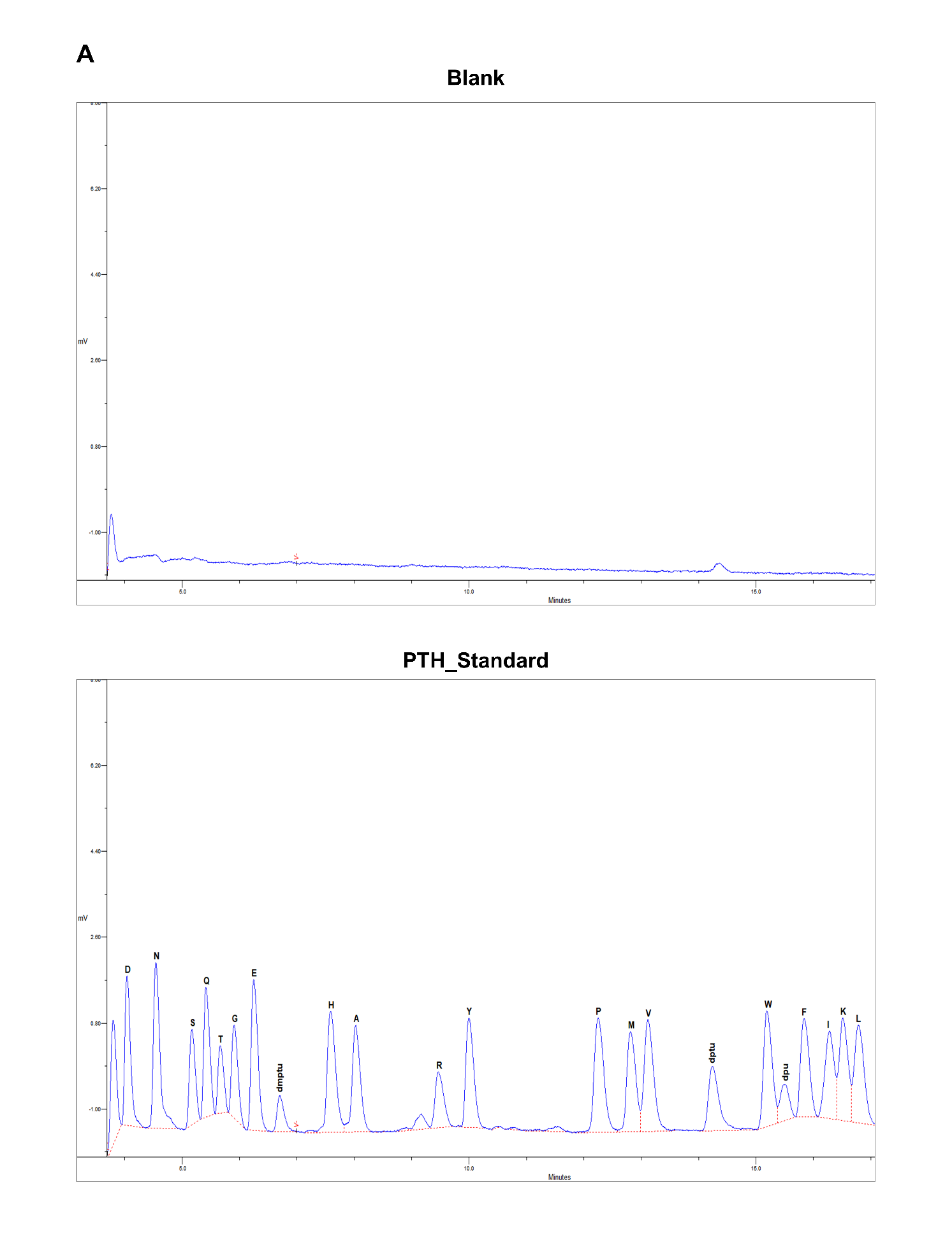


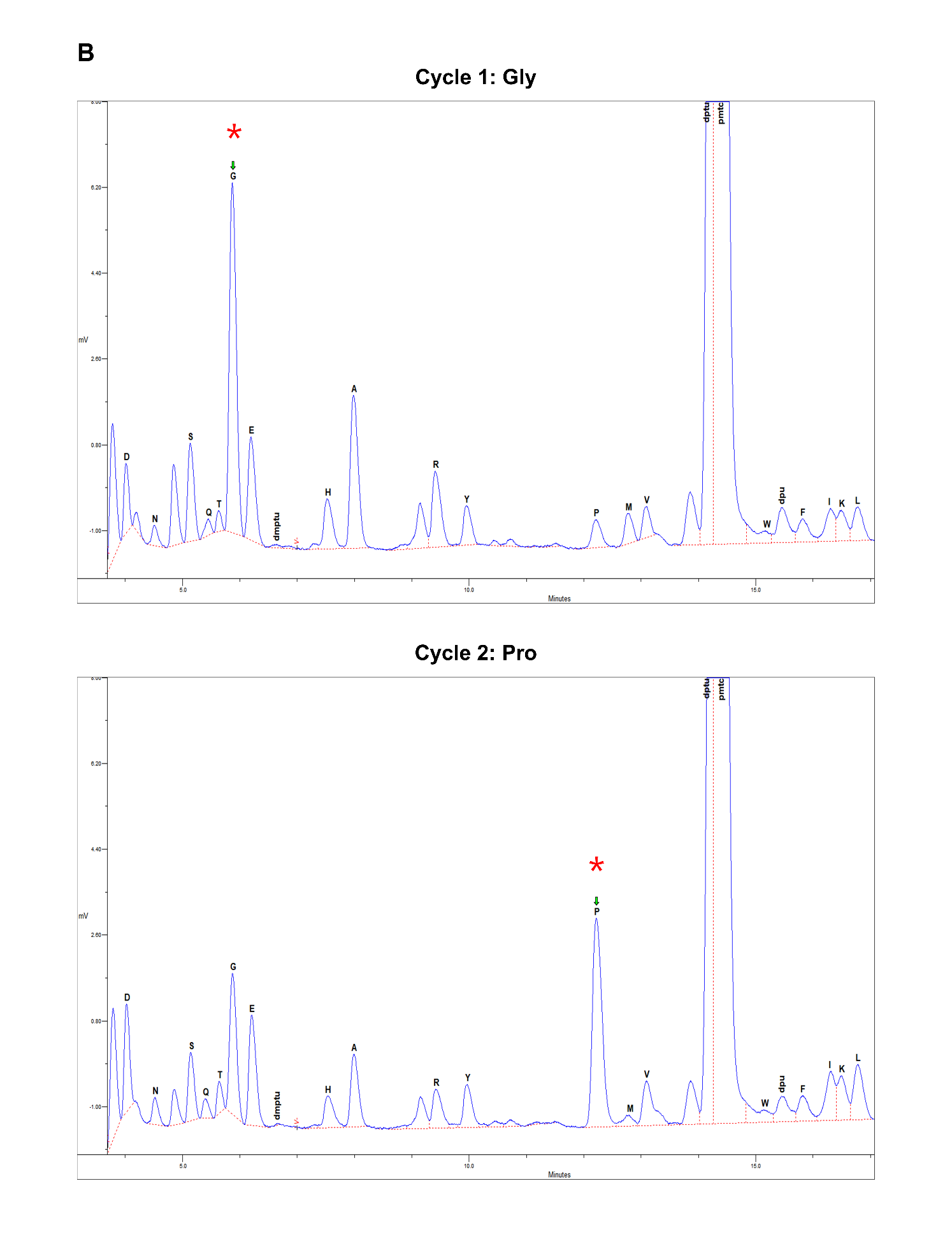


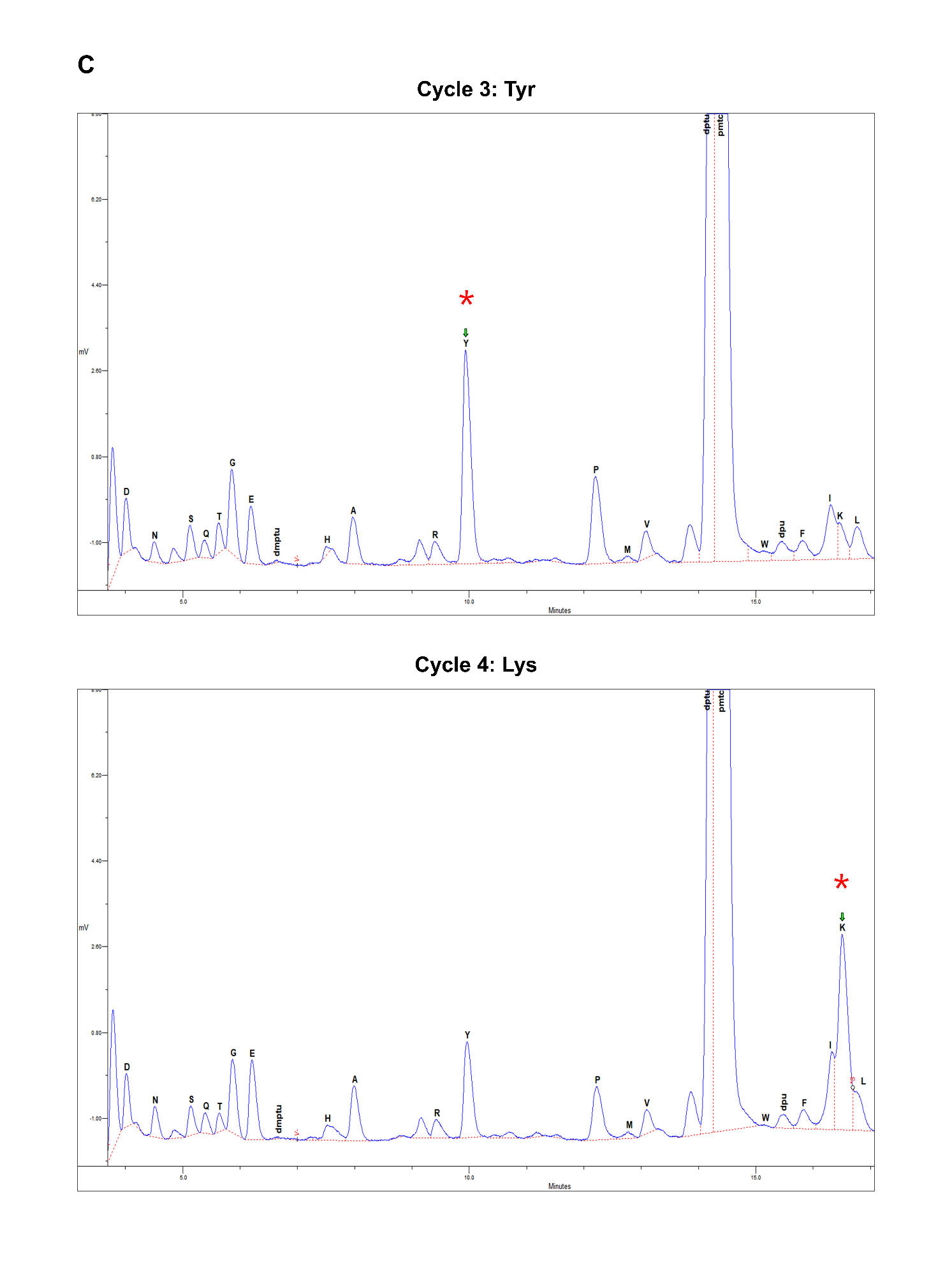


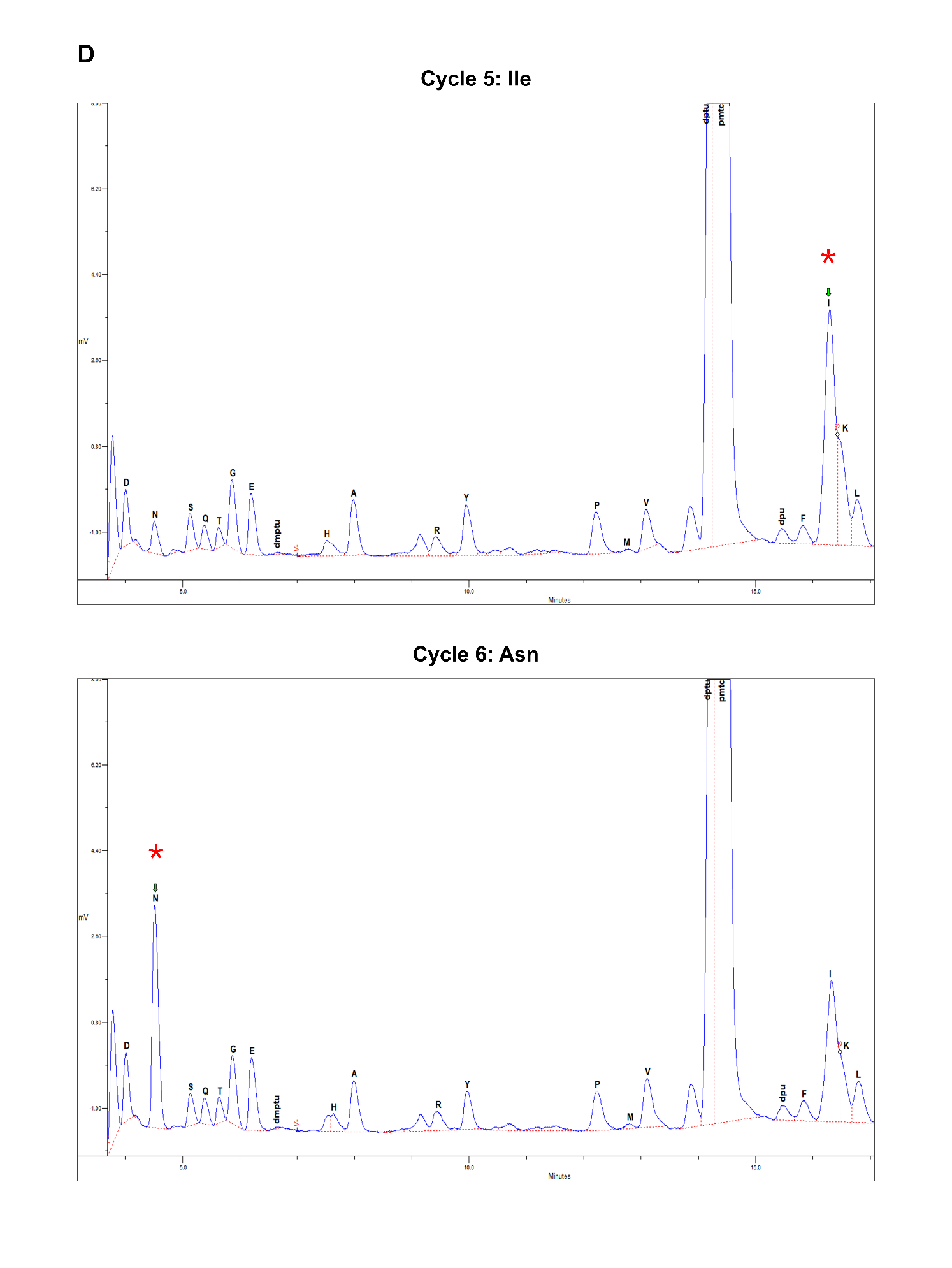


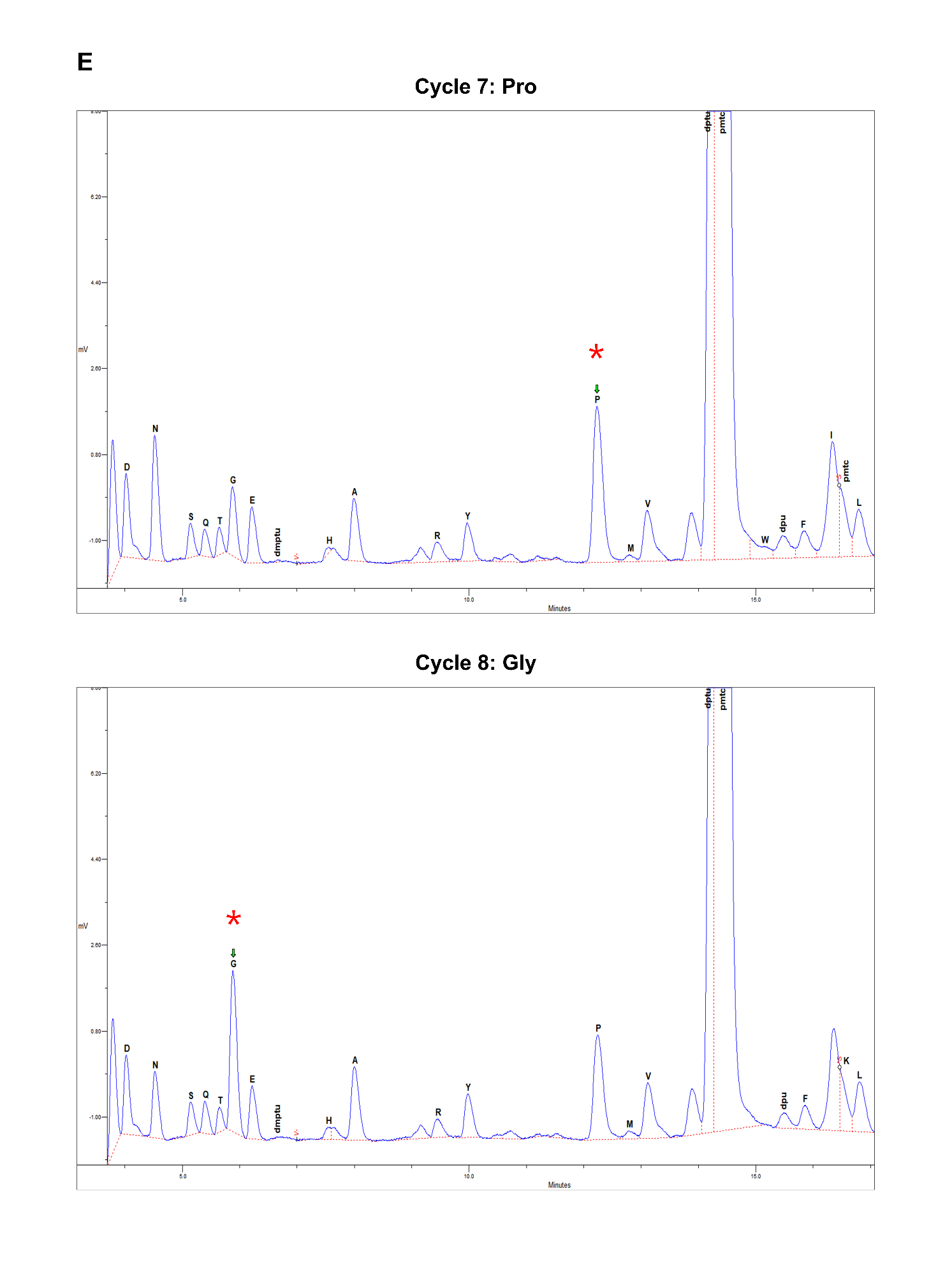


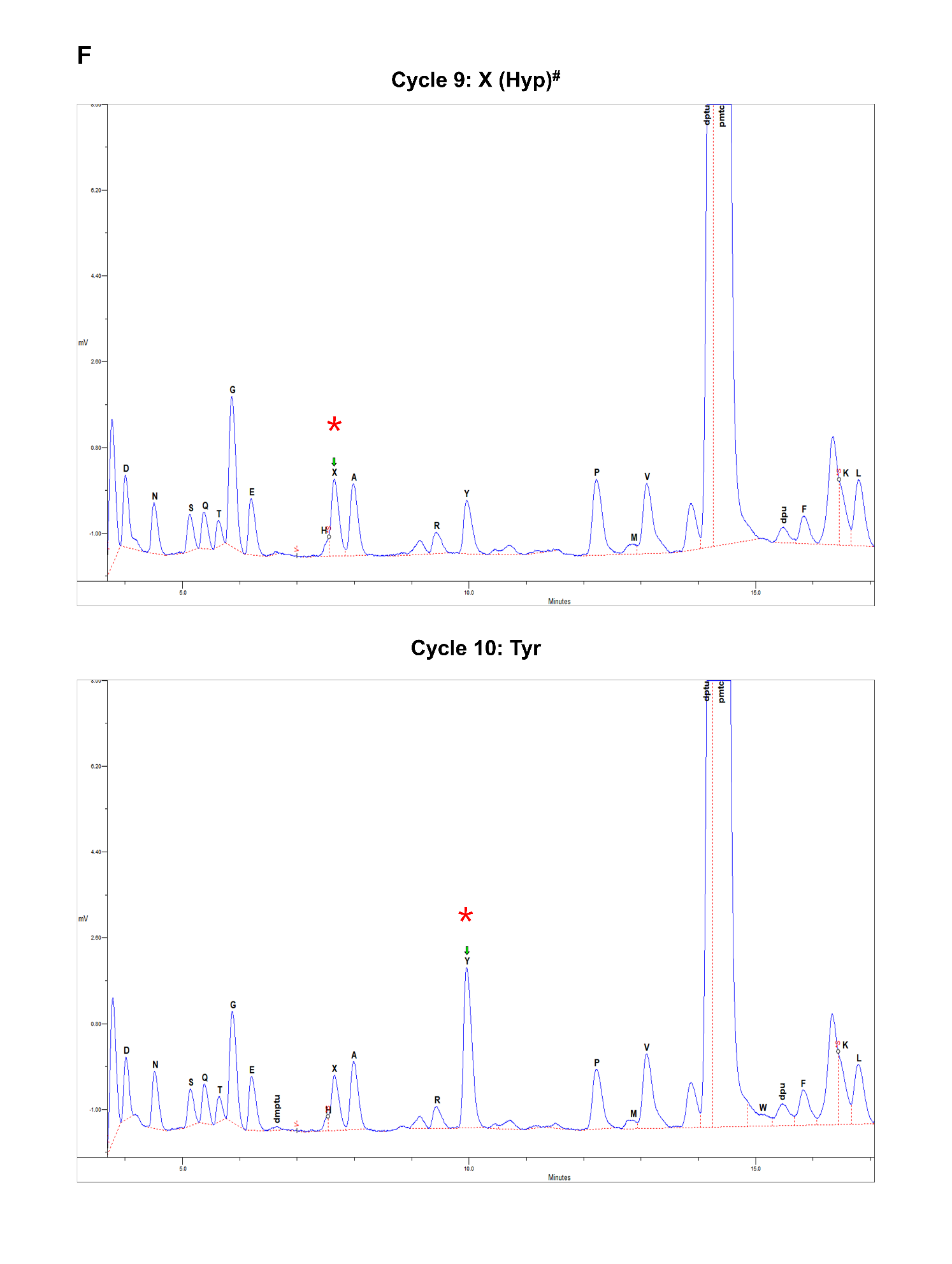


**Supplementary Figure S1.** **Phenylthiohydantoin (PTH) chromatogram from N-terminal sequencing by Edman degradation. A.** Chromatographic profiles of the blank and standard PTH derivatives used for sequencing calibration. **B−F.** Chromatographic profiles of PTH-amino acid derivatives from cycles 1 to 10. Asterisks indicate the positions of PTH-amino acids identified in cycle 1 to 10. The amino acid from cycle 9 was initially identified as hydroxyproline based on reference standards.


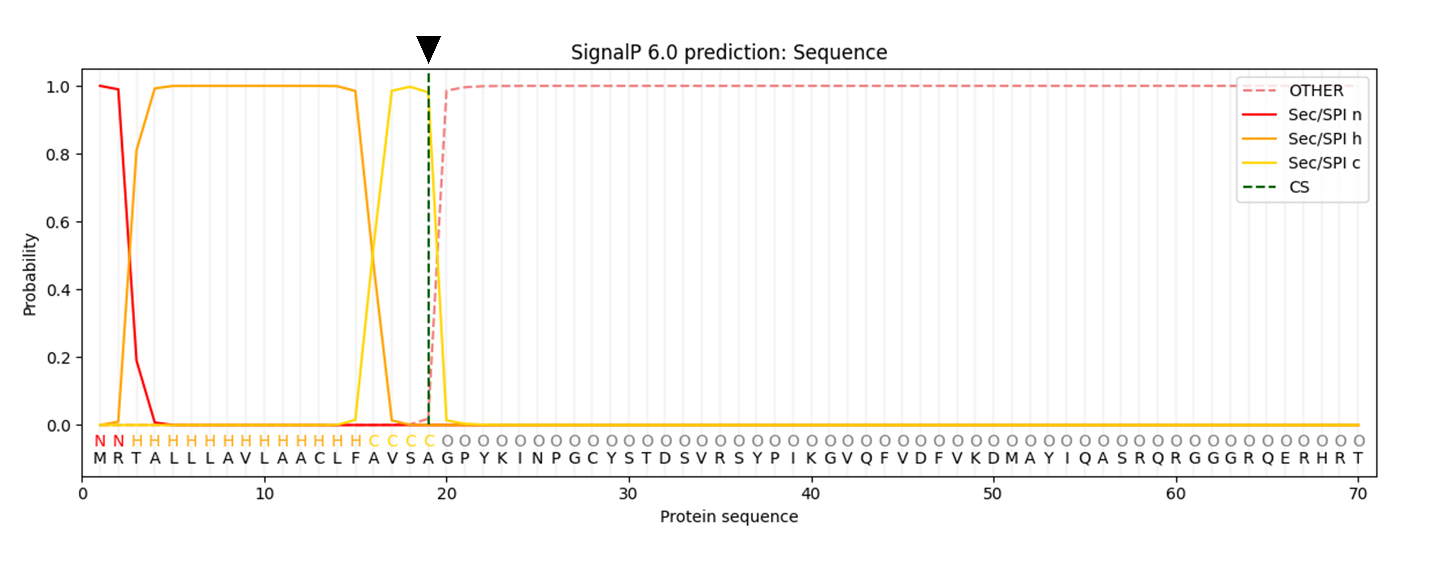


**Supplementary Figure S2.** **Signal sequence prediction**. Prediction of potential signal peptide region boundaries by SignalIP 6.0. The black triangle indicates the cleavage site between alanine (position 19) and glycine (position 20).

## Supplementary Table S1. Quantification of secreted proteins in *Chlorella* sp. HS2 culture medium.

| **Samples** | **Mean absorbance** | **Protein concentration**  **(mg mL^-1^)** |
| --- | --- | --- |
| Blank medium | 0.675 | 0.006558 |
| Secreted proteins^a^ | 0.898 | 0.401808 |
| Secreted proteins (1:4 dilution) | 0.744 | 0.128855 |
| Secreted proteins (1:20 dilution) | 0.688 | 0.029599 |

^a^The proteins secreted in the culture medium were concentrated 100-fold prior to analysis. Specifically, 10 mL of the culture supernatant was collected and precipitated with acetone. After precipitation, the protein pellet was resuspended in 100 µL of 1× phosphate-buffered saline (PBS) buffer, resulting in a 100-fold concentrated sample. The concentrated sample was additionally diluted 1:4 and 1:20 with 1× PBS buffer. Protein content was analyzed using the Pierce™ BCA Protein Assay Kits, and the absorbance was measured at 562 nm. A standard curve plotting the absorbance of the BSA standard was prepared according to the kit protocol and was used to determine the protein concentration of each sample. Blank medium served as a negative control.
